# Successful Conservative Treatment of Maternal Spontaneous Unilateral Adrenal Hemorrhage Causing Severe Anemia in the Third Trimester of Pregnancy—A Case Report

**DOI:** 10.3390/medicina60091448

**Published:** 2024-09-04

**Authors:** Tomasz Skołozdrzy, Jan Wojciechowski, Mirosław Halczak, Sylwester Michał Ciećwież, Maciej Ziętek, Maciej Romanowski

**Affiliations:** 1 Department of General and Oncological Surgery, Pomeranian Medical University, 70-111 Szczecin, Poland; 2 Department of Perinatology, Obstetrics and Gynecology Pomeranian Medical University in Szczecin, 72-010 Police, Poland

**Keywords:** pregnancy, adrenal hemorrhage, unilateral

## Abstract

We present the case of a 32-year-old pregnant woman in the 32 + 3 weeks of pregnancy who presented to the hospital with an exacerbation of pain in the right flank. The diagnostic evaluation revealed the presence of severe anemia and a spontaneous adrenal hemorrhage (SAH) in the right adrenal gland. The patient was transferred to the Perinatology, Obstetrics and Gynecology Clinic with the intention of undergoing preterm childbirth. However, the doctors made a risky decision to wait until week 37 and to terminate the pregnancy at that point. The decision was right, as a cesarean section was performed without complications, and the patient gave birth to a healthy child. Spontaneous adrenal hemorrhage (SAH) is a rare condition, defined as spontaneous hemorrhage without trauma or anticoagulant therapy. Due to bleeding and damage to the adrenal cortex, SAH can lead to adrenal insufficiency. Because of its non-specific symptoms and potentially fatal outcomes for the patient and fetus, it should be considered during diagnostics.

## 1. Introduction

A number of complications are frequently encountered during pregnancy. These include pregnancy-induced hypertension, obstetric cholestasis, gestational diabetes, and pathological bleeding. The causes of this latter condition include placental abruption and placenta previa. These dangerous complications are well documented in the medical literature, and due to the ease of imaging the bleeding, an accurate diagnosis can be made. However, due to the impact of pregnancy on a woman’s body, various pathologies may occur in almost any system, some of which may be overlooked but potentially fatal. One such pathology is a spontaneous adrenal hemorrhage (SAH), which is defined as a spontaneous hemorrhage associated with acute abdominal pain in the absence of prior trauma or anticoagulant therapy [1]. Autopsy reports indicate that the incidence of adrenal hemorrhage in unselected cases ranges from 0.03 to 1.8%; however, the prevalence among pregnant women remains unknown [2]. The etiology is believed to be an adrenal vascular rupture and central venous thrombosis [3]. The incidence of spontaneous adrenal hemorrhage typically involves the right adrenal gland [1].

Symptoms include the acute onset of pain in the lower back, abdomen, pelvis, or chest, nausea, and vomiting as well as hypotension. These manifestations are relatively common and are present in 65–85% of reported cases. However, these symptoms are non-specific; therefore, it is advisable to exercise caution.

The majority of cases are discovered incidentally; however, the presence of the aforementioned indicators is an important presenting symptom [4].

As a consequence of the damage to the adrenal cortex, a number of symptoms associated with adrenal insufficiency may be observed, including fatigue, weakness, dizziness, anorexia, nausea, vomiting, myalgia, and diarrhea [2,5].

In the physical examination, due to the retroperitoneal location of the adrenals, guarding, rigidity, and rebound tenderness may be observed [1].

The literature has identified a number of predisposing factors for this change, including the physiological hypervascularity of the adrenal glands, stress, cortical hypertrophy, hyperplasia, adrenal venous thrombosis, and hypertension [6,7]. Spontaneous adrenal hemorrhage may be caused by a number of obstetric factors, including antiphospholipid antibody syndrome, spontaneous abortion, postpartum hemorrhage, and torsion of an ovarian cyst [1].

The differential diagnosis of this condition includes an aneurysm of the renal artery, metastatic deposits in the adrenals, and renal tumors [4]. In pregnant women, right flank pain is most commonly associated with renal colic; however, it is also a presenting symptom in the majority of cases of non-hemorrhagic adrenal infractions [8].

The treatment of SAH is contingent upon the extent of the hemorrhage, the patient’s hemodynamic stability, and the presence of adrenal insufficiency. In challenging cases, surgical intervention may include traditional adrenalectomy. However, angiography and embolization of the bleeding lesions have been proposed as a potentially more efficacious approach. In cases where the patient is clinically stable, conservative treatment is typically employed, comprising steroid therapy, serial imaging, and close monitoring [4].

This case report presents a rare instance of spontaneous adrenal hemorrhage in a 32-year-old pregnant woman who was successfully managed with a conservative approach.

## 2. Case Description

A 32-year-old nulliparous patient with a gestational age of 32 + 6 weeks was referred from a community hospital to the Department of Perinatology, Obstetrics and Gynecology due to the presence of bleeding within the capsule of the right adrenal gland.

(a) Treatment in the community hospital

The patient presented with a history of increasing pain on the right side of the back and was admitted to the community hospital at 32 + 3 weeks with a clinical suspicion of renal colic on the right side.

The patient had previously experienced an ectopic pregnancy in the fallopian tube in the third week of the first trimester of pregnancy, which was treated with salpingectomy. No history of previous diseases or surgical procedures was noted. A spontaneous pregnancy was confirmed, and the patient denied any issues with conception. The patient was administered standard doses of folic acid and Tardyferon with the objective of preventing iron deficiency during pregnancy. The patient had not previously undergone any treatment involving antiaggregants or anticoagulants. Furthermore, there was no history of trombophilic or pro-hemorrhagic polymorphisms. Additionally, these pathologies were not present in the patient’s family history.

One month prior to the onset of abdominal symptoms, the patient presented with dizziness and elevated blood pressure (BP 140/90 mmHg), which was accompanied by headaches and nasal bleeding. However, these symptoms abated without any form of intervention.

A physical examination revealed some deviations. The patient presented with pain in the right lumbar region. Furthermore, she exhibited signs of anxiety. At the community hospital, the patient began vomiting dark-colored vomit in large amounts.

A decreased baseline variability in the fetal heart rate pattern was observed during the cardiotocographic examination. The basic laboratory findings indicated severe anemia and the CRP level was elevated. The patient exhibited tachycardia at a rate of approximately 120 beats per minute and blood pressure below the norm (100/60 mmHg), which is indicative of hemorrhagic shock (Table 1).

An abdominal ultrasound was conducted, which revealed the presence of a hemorrhagic lesion in the right adrenal gland with an approximate measurement of 110 × 63 × 130 mm. The lesion was hypoechogenic in comparison to hepatic parenchyma. Further diagnostic procedures were undertaken, including an MRI scan, which revealed areas with a high T1WI signal, indicative of the presence of hemorrhagic lesions. The bleeding was observed to be propagating along the right renal fascia.

Six units of red cell concentrate and four units of fresh frozen plasma were transfused. Additionally, fluid therapy was initiated. Given a C-reactive protein (CRP) level of 60 mg%, the patient was initiated on empirical antibiotic therapy. Additionally, intravenous hydrocortisone was administered, and prenatal corticosteroid therapy in the form of betamethasone was initiated to facilitate fetal lung maturation. A surgical consultation was conducted, during which the termination of the pregnancy within a two-to-three-day timeframe was recommended. Additionally, adrenalectomy was proposed as a potential course of action should bleeding recur.

The patient was subsequently transferred to the Department of Perinatology, Obstetrics and Gynecology, given the department’s superior experience in managing preterm births in patients with severe complications.

(b) Treatment in the Clinic

The patient was admitted to the clinic, and based on information provided by the community hospital, doctors kept the idea of starting childbirth within a few days in mind. A multidisciplinary consultation was conducted at the hospital involving a surgeon and an endocrinologist. In the endocrinological consultation, the gradual discontinuation of hydrocortisone supplementation, the indication of metoxycatecholamine levels in 24 h urine collection, and the indication of adrenal hormones were advised. The endocrine surgeon was apprised of the possibility of performing an adrenalectomy during the parturition, given the patient’s case.

**Figure 1:**
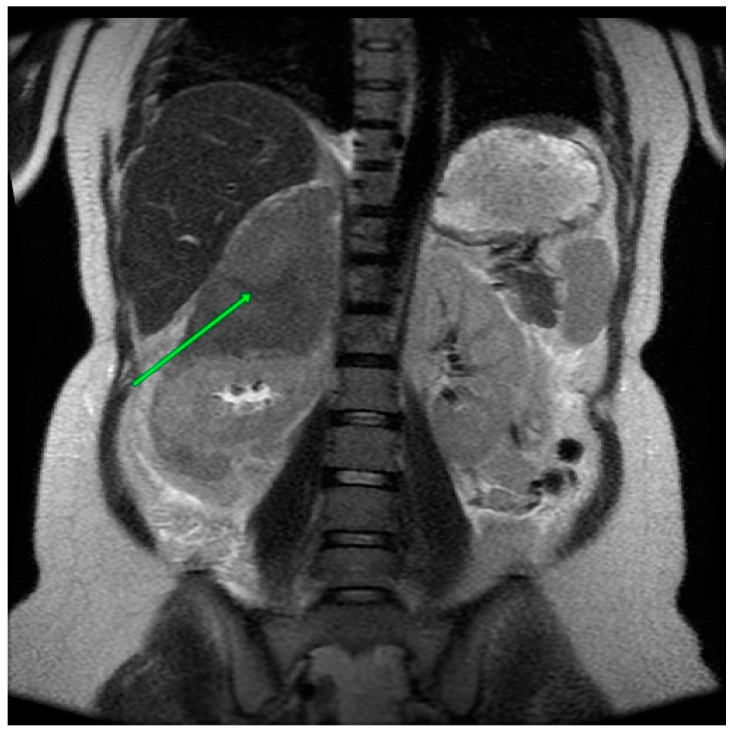
An abdominal MRI, revealed a hematoma in the right adrenal gland.

Imaging was conducted in the clinic, commencing with an ultrasound. The imaging results demonstrated the presence of a heterogeneous lesion in the projection of the right adrenal gland. The lesion exhibited a mixed echogenicity and measured 123 × 59 × 53 mm. A Doppler scan revealed no vascularity. Four days later, an MRI scan was conducted. The scan revealed the presence of a hematoma in the right adrenal gland with dimensions of 12 × 7.3 × 6.7 cm. The lesion exhibited a heterogenous, mixed-signal in both T1- and T2-weighted images. The lesion compressed and displaced the right kidney. A solid mass measuring 2.1 cm in width was identified in the vicinity of the right kidney, and it was suspected to be a hematoma. Furthermore, a thin smudge of blood was identified along the lower edge of the liver. Additionally, a minor hydronephrosis was observed in the vicinity of both kidneys (Figure 1).

Following the administration of red cell concentrate and fresh frozen plasma at the community hospital, the patient’s blood count demonstrated a notable improvement, with a total red blood cell count of 3.05 × 10^6^/μL, a hemoglobin concentration of 9.6 g/dL and a hematocrit level of 28.5%. A physical examination revealed that the patient continued to experience pain in the right lumbar region. However, the patient’s anxiety levels subsequently decreased. Her blood pressure was recorded at 125/80 mmHg with a heart rate of approximately 80 beats per minute. The patient’s parameters remained stable throughout the course of her hospitalization.

The initial hormonal tests conducted during the patient’s initial days of hospitalization demonstrated that the majority of the observed parameters were within the normal range. The only observed deviations were in the cortisol level with lowered ACTH due to the ongoing hydrocortisone supplementation. However, following a reduction in dosage and subsequent cessation of treatment, the parameters returned to within the normal range (Table 2).

Imaging did not reveal any evidence of recent bleeding. The patient’s condition remained stable, and the results of the hormonal panels fell within the normal range. This prompted perinatologists to reconsider initiating pre-term delivery, given the heightened risk for complications in the infant. A decision was made to observe the patient with the option of initiating labor if her condition deteriorated. This proved to be a correct decision as her pain gradually subsided over the following weeks, and there were no further signs of fresh bleeding.

The patient was treated conservatively until the elective termination of the pregnancy at week 37.

A decision was taken to perform a cesarean section in accordance with the Misgav-Ladach method. The uterine muscle was incised transversely at the lower segment for a length of 3 cm and subsequently stretched laterally without the utilization of a surgical knife. The amniotic fluid was observed to be clear. A female neonate was born in good condition overall. There was no evidence of uterine bleeding or pathological changes in the adnexa, uterus, or pelvic organs. The patient was hemodynamically stable. The surgeon elected to withdraw from the planned removal of the right adrenal gland.

**Figure 2:**
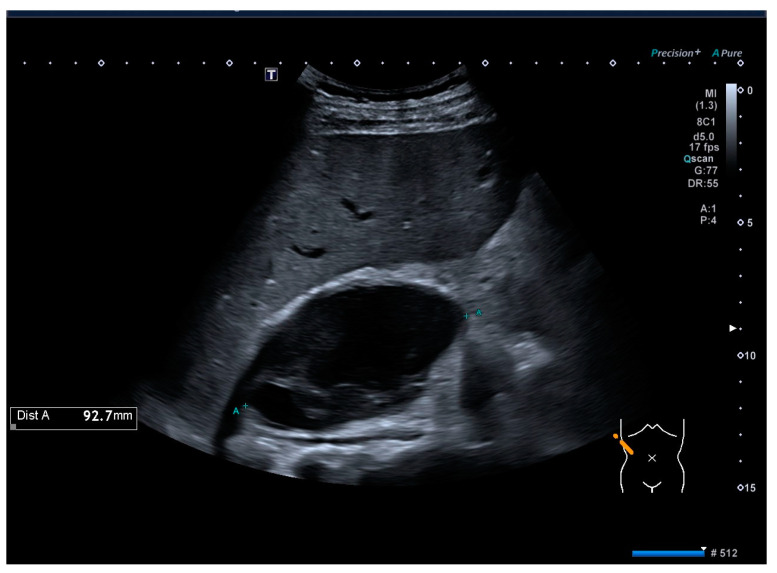
Abdominal ultrasound. A hematoma is visible in the projection of the right adrenal gland.

A further ultrasound was performed the following day. It demonstrated the presence of a partially liquefied hematoma in the projection of the right adrenal gland, with a measured volume of 93 × 56 × 63 mm (Figure 2).

The patient was discharged from the hospital three days after the procedure, exhibiting no signs of illness or complications. A decision was made to schedule an outpatient surgical consultation in two months’ time due to the presence of a hematoma in the right adrenal gland.

The scheduled consultation was conducted as planned. The patient did not report any complaints. The physical examination yielded no evidence of peritoneal symptoms or pathological resistance. The patient was advised to undergo a CT scan in three months’ time.

**Figure 3:**
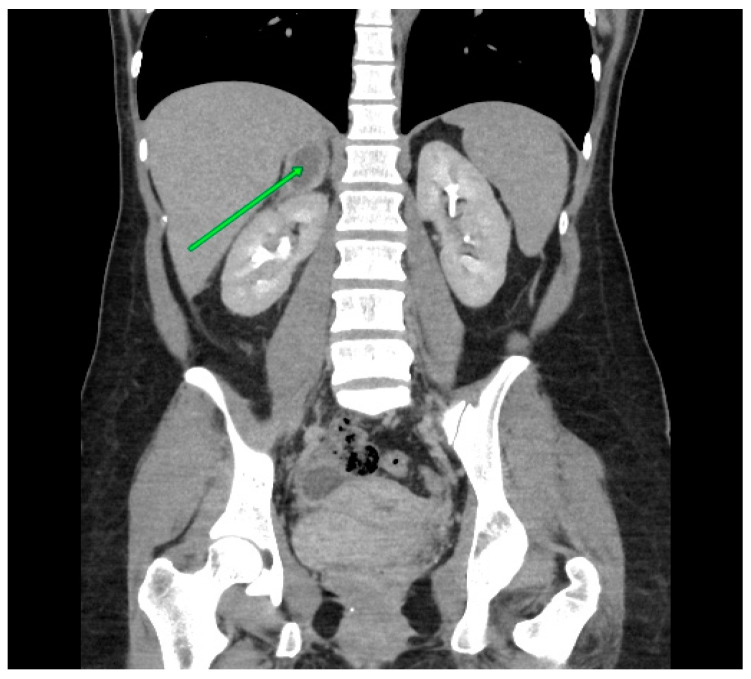
Abdominal CT scan. A hematoma is present in the projection of the right adrenal and is markedly smaller in size than that observed in the MRI performed three months ago.

A control CT was performed three months after the cesarean section. The CT scan demonstrated a reduction in the size of the hematoma, which was measured at 28 × 23 × 11 mm (Figure 3). The patient continues to be managed by an endocrine surgeon.

## 3. Discussion

Lower back pain is a prevalent condition among pregnant women. Studies estimate that 50% of pregnant women experience this condition, with the rates ranging from 25% to 90% [9]. This presentation in our patient could have been readily misdiagnosed as a pelvic girdle or lumbar pain. Our case demonstrates that even symptoms that are not immediately life-threatening may be indicative of a potentially fatal disease, such as a spontaneous adrenal hemorrhage.

The majority of patients present with no deviations from baseline laboratory findings; however, leukocytosis and anemia may be present [4]. This was the case with our patient, resulting in hemorrhagic shock. In pregnant women, it is important to consider the potential for alterations in laboratory test reference ranges due to the physiological changes associated with pregnancy.

In the event of adrenal insufficiency, it is possible for hyponatremia, hyperkalemia, and hypoglycemia to occur. As a consequence of transient release from the gland in the event of destruction, excess cortisol and elevated catecholamine levels might be observed [4]. In cases where there is a suspicion of adrenal gland damage, it is crucial to order endocrine panels to assess the patient’s morning cortisol and morning ACTH levels. The gold standard for diagnosing adrenal gland damage is a cosyntropin or standard-dose ACTH stimulation test [10].

It is also important to exclude thrombophilias, such as the antiphospholipid syndrome, as potential etiologies of adrenal vein thrombosis and a subsequent hemorrhage through the measurement of platelet count and coagulation parameters. In the case of our patient, the aforementioned parameters were within the normal range [2].

Pregnancy is associated with an elevated risk of venous thromboembolism (VTE), encompassing deep vein thrombosis (DVT) and pulmonary embolism (PE). The risk is approximately five to twenty times higher than that observed in non-pregnant women of a similar age [11,12]. In accordance with the guidelines set forth by the American College of Obstetricians and Gynecologists, heparin compounds are the preferred anticoagulants during pregnancy. This is due to the fact that they do not cross the placenta and are safe for the fetus. However, the use of these agents can result in complications such as bleeding, which can potentially lead to serious adverse events such as SAH. Heparin agents employed during pregnancy are divided into two categories: unfractionated heparin (UFH) and low-molecular-weight heparin (LMWH). LMWH has a superior safety profile and a lower incidence of adverse effects, including bleeding, heparin-induced thrombocytopenia (HIT), or heparin-associated osteoporosis, which are lower [13].

In the systematic review conducted by Simard et al., the incidence of major bleeding events was found to range between 2.9% and 5.0% in women receiving therapeutic anticoagulation for an acute VTE during pregnancy [12]. However, it is noteworthy that the majority of these events were postpartum hemorrhages. The most common cause of VTE in pregnancy is inherited thrombophilia, which encompasses a range of genetic polymorphisms like the factor V Leiden (FVL) mutation, prothrombin G20210A mutation, and deficiencies of protein C (PC), protein S (PS), and antithrombin (AT), as well as methylenetetrahydrofolate reductase enzyme (MTHFR) 1298 and 677 mutations, and the Plasminogen activator inhibitor-1 (PAI-1) mutation [14,15,16]. It is, however, noteworthy that a recent study by Sokol Karadjole et al. demonstrated that the patients being asymptomatic carriers of inherited thrombolytic polymorphisms do not have an increased risk of adverse perinatal outcomes [16].

Ultrasound is typically employed for initial diagnosis; however, a smaller adrenal mass might be challenging to evaluate, particularly in the left adrenal gland. Moreover, sonographic features are non-specific in identifying such masses [17]. A further limitation of ultrasound imaging is the difficulty in accessing deeper-lying soft tissue structures within the abdominal cavity and pelvis.

Magnetic resonance imaging (MRI) is the most accurate imaging technique in SAH, as it can differentiate between subacute and chronic hemorrhage [6]. In non-pregnant patients, contrast-enhanced CT is also employed [1]. In a CT scan, adrenal hemorrhage presents as a round or ovoid lesion. In some cases, peri-adrenal fat stranding may be visible, as observed in our patient, and bleeding may extend into the perinephric space [4]. In addition, slight hydronephrosis was observed in this patient. The presence of fresh hematomas is indicative of high attenuation, which subsequently diminishes over time [4].

Nevertheless, during pregnancy, the use of CT scans entails a considerable risk of fetal radiation exposure, and therefore, they should be employed with caution and only when absolutely necessary. Ionizing radiation has been demonstrated to exert a detrimental impact on the developing fetal brain. In the event of exposure occurring between the 8th and 15th weeks of gestation, the fetus is at risk of developing microcephaly or an intellectual disability (exposure of 60–310 mGy) [18]. Furthermore, there is an increased risk of developing pediatric cancers, particularly hematological malignancies (exposure of 10–20 mGy) [9]. For comparison, a low-dose abdominal CT is related to fetal radiation between 1.5 and 35 mGy, while the corresponding dose for a pelvic CT varies between 10 and 50 mGy [19]. In conclusion, it is not advisable to perform CT scans during pregnancy.

The management of SAH in pregnancy is contingent upon the patient’s stability. SAH in pregnancy may be managed conservatively, particularly following an evaluation of adrenal function and the use of diagnostic imaging techniques such as MRI [6]. Conservative treatment methods include steroid therapy, fluid resuscitation, pain management, the maintenance of hemodynamic stability, and correction of underlying coagulopathies or comorbidities that may exacerbate the symptoms [10].

In patients with hemodynamic instability, preterm delivery and emergency adrenalectomy may be indicated. However, in the case presented, the patient’s condition was stable, and these procedures were, therefore, not required [1]. In the event of a severe hemorrhage, arterial embolization may be required as a temporary measure prior to subsequent surgical intervention [5]. It is imperative to consider the potential for bilateral SAH in patients who have experienced unilateral SAH [20].

There is a paucity of studies in the literature on the optimal mode of delivery in patients with SAH [2]. However, if the patient is stable, vaginal delivery can be undertaken safely. Nevertheless, in our patient’s case, the decision was taken to perform a cesarean section in accordance with the Misgav-Ladach method. This method gained popularity at the end of the 20th century and is characterized by a reduction in the amount of suture material used, the use of blunt preparation techniques, and the avoidance of sharp cuts other than on the skin and uterus.

The Misgav-Ladach approach starts with a scalpel incision of the skin and subcutaneous tissue approximately 2 cm in length, reaching the fascia of the rectus abdominis muscle. Subsequently, the skin, subcutaneous tissue, and other tissues are prepared using a blunt technique. The uterine muscle is incised transversely in its lower section, and the uterine cavity is opened bluntly, spreading the wound sideways. The uterine muscle and fascia are then sutured with a continuous suture without shuffling. The peritoneum, rectus abdominis muscles, and subcutaneous tissue are left unstitched. The skin is then sutured with an intradermal suture.

This approach yielded numerous advantages, including a reduction in perioperative morbidity with a significant reduction in postoperative early and late complications and a reduction in the use of analgesics, antibiotics, and blood products. Parturient women exhibited enhanced recovery and recuperation, accompanied by a discernible reduction in overall treatment costs. A substantial body of clinical evidence has proven all of these benefits [21].

The reduction in suture material, the use of blunt preparation, and the avoidance of sharp cuts other than on the skin and uterus all contributed to a reduction in perioperative morbidity with a significant reduction in postoperative early and later complications. Furthermore, the use of analgesics, antibiotics, and blood products resulted in better and faster recovery of parturient women and an overall reduction in treatment costs. These findings have been corroborated by numerous clinical studies. Nevertheless, the Misgav-Ladach method cannot be considered the gold standard in performing cesarean sections in patients with SAH, given that it is not evidence-based.

Furthermore, it is recommended that adrenal function be assessed postpartum, as the stress associated with labor has the potential to precipitate a relapse [6]. The most significant consequence of an adrenal hemorrhage is the development of adrenal insufficiency, which may be transient or permanent. In the event of extensive bleeding, there is a risk of hypovolemic shock [4]. A follow-up MRI or CT scan is recommended to confirm whether the hematoma has stabilized or resolved [2]. It is imperative to evaluate adrenal function, as fatal adrenal insufficiency secondary to SAH has been documented in postpartum patients [6].

The data were gathered from eight publicly accessible studies, which collectively described nine patients with an incidence of SAH during pregnancy (Table S1) [2,3,5,7,10,22,23,24]. In all but one instance, patients were admitted to the hospital between 32 and 39 weeks of gestation, which aligns with the case of our patient. The sudden onset of the flank pain was a distinctive feature of the condition. Only one publication reported a previous uneventful pregnancy, which is consistent with the case of our patient. Anemia was reported in four cases, with one instance being severe. Four cases reported hormonal deviations, with one instance of adrenal insufficiency. Four patients underwent cesarean-section delivery, while three underwent spontaneous labor. The imaging modalities employed for lesion diagnosis included ultrasound in three patients, CT in five patients, and MRI in five patients. Two patients experienced premature birth. In two cases, the surgical procedure of adrenalectomy was performed.

A comparison of the previously referenced cases with our occurrence of severe anemia and hemorrhagic shock has only been described in the research by Imga et al. [7] In contrast to our case, the authors did not await the outcome and instead performed a cesarean section in the same week, in addition to which they also performed an adrenalectomy. To the best of our knowledge, this represents the first case of SAH complicated with hemorrhagic shock that has been treated conservatively. The case study by Imga et al. shows that the obstetricians and surgeons from our hospital assumed a degree of risk by postponing the delivery. However, this decision ultimately proved beneficial, as the patient went on to undergo labor in term and was spared from the necessity of undergoing adrenalectomy.

## 4. Conclusions

Non-traumatic adrenal hemorrhaging represents a rare but potentially fatal complication of pregnancy. If left undiagnosed and untreated, SAH is associated with poor maternal and fetal outcomes. In the case of our patient, SAH was complicated with severe anemia and hemorrhagic shock. For the majority of obstetricians, this would be a reason to terminate the pregnancy as soon as possible. However, our experience demonstrates that if the patient is treated in a highly specialized obstetric department, conservative treatment can be employed and prolonged until the recommended childbirth time range.

## Figures and Tables

**Table 1:** The patient’s baseline laboratory findings from the community hospital.

Parameter	Level
Hemoglobin	6.9 g/dL
Hematocrit	21.3%
RBC	2.07 × 10^6^/μL
MCV	95.9 fL
MCH	33.3 pg
MCHC	34.7 g/dL
PDW	12.9 fL
WBC	14.3 × 10^3^/μL
PLT	11.4 × 10^3^/μL
CRP	61.1 mg/L
Potassium	3.02 mmol/L
Natrium	142 mmol/L
Procalcitonin	0.3 ng/mL

**Table 2:** The patient’s hormonal laboratory findings from hospitalization in the clinic.

Parameter	Level
ACTH (while supplementing hydrocortisone)	1.59 pg/mL
Cortisol (while supplementing hydrocortisone)	30.3 μg/dL
ACTH	27.6 pg/mL
Cortisol	9.7 μg/dL
Androstendione	0.91 ng/mL
Testosterone	0.243 ng/mL
17-hydroxyprogesterone	5.69 ng/mL
DHEA	6.5 ng/mL

## Data Availability

The original contributions presented in this study are included in the article, further inquiries can be directed to the corresponding author.

## Supplementary Materials

The following supporting information can be downloaded at: https://www.mdpi.com/article/10.3390/medicina60091448/s1, Table S1: List of studies, which collectively described patients with an incidence of SAH during pregnancy.
