# Supplementary material for: Successful Conservative Treatment of Maternal Spontaneous Unilateral Adrenal Hemorrhage Causing Severe Anemia in the Third Trimester of Pregnancy—A Case Report
Source: Medicina (Kaunas). 2024 Sep 4;60(9):1448. doi: 10.3390/medicina60091448 (PMC11434074; doi:10.3390/medicina60091448)
Supplement: Supplementary file 1 [file medicina-60-01448-s001.zip › medicina-3187299-supplementary.pdf]

Table S1: List of studies, which collectively described patients with an incidence of SAH during pregnancy.

**Publication**	**Week of pregnancy in which the patient came to the hospital**	**Symptoms**	**Previous pregnancy history**	**Laboratory panels deviations**	**Imaging methods used to diagnose**	**Week and type of delivery**	**Time from beginning of hospitalization to delivery**	**Adrena-lectomy**
Kadhem et al. [5]	36 week	Left flank, constant sharp pain which was getting worse. Nausea, vomiting and fatigue	Two uneventful pregnancies	Low morning cortisol level	Ultrasound, MRI	36 week, vaginal	2 days	No
Gupta et al. [2] Patient 1	36 week	Sudden onset of left sided pain, gestational diabetes, elevated blood pressure	N/A	Within the norm	Ultrasound, CT	39 week, vaginal	3 weeks	No
Gupta et al. Patient 2	38 week	Sudden onset of the left upper quadrant pain that radiated to the midline	Two elective delieveries	Low ACTH with normal cortisol level	CT, MRI	38 week, vaginal	Patient admitted in spontaneous labour	No

Desai et al.[10]	33 week	Abdominal pain radiating to the left side, nausea, emesis, chest pain, shortness of breath	One childbirth	Within the norm	CT	39 week, cesarean section	6 weeks	No
Yang et al. [3]	17 week	Tenderness in the left epigastrum	N/A	Slightly lowered Hemoglobin and RBC, elevated norurinenephrine, dopaminę, 24-hour urinary free cortisol and blood norepinephrine	MRI	Non specified	Non specified	Yes
Imga et al. [7]	34 week	Left epigastrum pain, right flank pain, tachypnea, cold sweats, elevated blood pressure, then hemorrhagic shock	N/A	Hemoglobin- 6.9 g/dl	MRI	34 week, cesarean section	Non specified, in the same week	Yes
Singh et al. [17]	38+6 week	Right loin pain, increased blood pressure, fever	N/A	Hemoglobin- 8.9 g/dl	Ultrasound, MRI	39 week, Cesarean Section	2 days	No

		with chills, dysuria						
Almutairi et. al. [18]	36+4 week	Acute,severe non-radiating abdominal pain located at right lower quadrant area , nausea	Three deliveries	Mild anemia- 9.6 g/dl	CT	39 week, cesarean section	Over 2 weeks	No
Songtanin et. al. [11]	32 week	Sudden onset left flank pain with nausea	N/A	Low cortisol, adrenal insufficiency in laboratory panels	CT	Non specified	Non specified	No
